# A Simplified Method of Synthesis to Obtain Zwitterionic Cellulose under Mild Conditions with Active Ionic Moieties

**DOI:** 10.3390/molecules25133065

**Published:** 2020-07-05

**Authors:** Nadia B. Haro-Mares, Juan C. Meza-Contreras, Fernando A. López-Dellamary Toral, Ricardo González-Cruz, José A. Silva-Guzmán, Ricardo Manríquez-González

**Affiliations:** Department of Wood, Cellulose and Paper, CUCEI, University of Guadalajara, 45020 Guadalajara, Jalisco, Mexico; aradia_b@hotmail.com (N.B.H.-M.); jcmezac@academicos.udg.mx (J.C.M.-C.); ferdellam@gmail.com (F.A.L.-D.T.); ricardo.gonzalezc@academicos.udg.mx (R.G.-C.); jasilva@dmcyp.cucei.udg.mx (J.A.S.-G.)

**Keywords:** zwitterionic cellulose, N-protected aspartic anhydride, functionalization degree, amino acid deprotection, mild conditions, ionic moieties

## Abstract

A simplified procedure to synthesize zwitterionic cellulose by means of N-protected aspartic anhydride under mild conditions was developed. The preparation of modified cellulose samples was carried out under heterogeneous, aqueous conditions by reacting NH_4_OH-activated cellulose with aspartic anhydrides N-protected with trifluoroacetyl (TFAc) and carbobenzyloxy (Cbz). Modified cellulose samples Cel-Asp-N-TFAc and Cel-Asp-N-Cbz were characterized by Fourier Transform Infrared (FTIR) and ^13^C solid state Nuclear Magnetic Resonance (NMR) spectroscopy. The functionalization degree of each cellulose sample was determined by the ^13^C NMR signal integration values corresponding to the cellulose C1 vs. the Cα of the aspartate residue and corroborated by elemental analysis. In agreement, both analytical methods averaged a grafting degree of 20% for Cel-Asp-N-TFAc and 16% for Cel-Asp-N-Cbz. Conveniently, Cel-Asp-N-TFAc was concomitantly partially N-deprotected (65%) as determined by the ninhydrin method. The zwitterion character of this sample was confirmed by a potentiometric titration curve and the availability of these amino acid residues on the cellulose was inspected by adsorption kinetics method with a 100 mg L^−1^ cotton blue dye solution. In addition, the synthesis reported in the present work involves environmentally related advantages over previous methodologies developed in our group concerning to zwitterionic cellulose preparation.

## 1. Introduction

Cellulose is the most abundant structural component in plants, i.e., 10% to 20% of leaf dry weight, almost 50% in wood and bark and 90% in cotton linters. It is also one of the most abundant natural polymers in the world, easy to get, economically profitable, biodegradable, renewable and has attractive mechanical, biological and chemical properties [1]. From a chemical point of view, cellulose is a polysaccharide composed of linear chains of anhydroglucose repeating units (AGU) linked together through β 1→4 glycosidic bonds. As a polysaccharide, hydroxyls (-OH) are the main functional group, which are suitable for chemical modification by means of substitution, addition and oxidation reactions [2,3,4]. Many of these chemical modifications take place in homogenous reactions where cellulose is solubilized under different chemical conditions in order to increase the degree of substitution and improve the distribution of the chemical groups along the polymer [5,6]. However, such processes involve large amounts of organic solvents, both during the reaction and for the purification of the products. Moreover, most of the solvents and chemicals typically used are toxic and harmful to the environment, i.e., CS_2_, dimethylacetamide, lithium chloride, urea, concentrated acids and alkalis among others [7,8,9,10]. On the other hand, chemical modification of cellulose under heterogeneous reactions has been another possibility to introduce different chemical functionality onto the surface. This grafting procedure takes place when cellulose stays insoluble during the modification process. Although the degree of substitution in the polysaccharide is lower in comparison with the homogenous reaction, the amount of harmful waste produced by heterogeneous reactions can be also lower or in some cases solvent free [11]. Thus, both procedures present advantages and disadvantages in relation to achieving better polymer properties or promoting environmentally friendly procedures.

An area of interest in cellulose modification research has been to confer, for different purposes, ionic characteristics to this polysaccharide, by means of introducing cationic or anionic moieties [12,13,14,15]. On the other hand, some reports have explored the modification of cellulose using zwitterionic groups, as well as its potential uses and applications [16,17,18,19]. In this regard, investigations by some members of our group have previously proved that covalently attaching amino acids (with equimolar amount of negative and positive sites) on cellulose fibers contributes in increasing the interfiber bonding strength around 35% in wet paper sheets [20]. Accordingly, solid state of ^13^C and ^15^N Nuclear Magnetic Resonance (NMR) spectroscopy has been employed for the characterization of the zwitterionic cellulose and determining how the zwitterionic groups are arranged. Furthermore, zwitterionic interactions between amino acid residues on cellulose fibers were also investigated with the measurement of internuclear ^13^C–^15^N distances by solid state NMR employing Rotational-Echo Double-Resonance (REDOR) experiment [21]. Consequently, these results motivated us to inquire into possible applications, such as adsorption and separation systems for ions (i.e., heavy metal uptake in polluted water and ionic chromatography), new adhesives, and enzyme and cell immobilization for use in biocatalysis (recoverable catalytic systems). However, the preparation method that we first developed for this zwitterionic cellulose [22] is not environmentally friendly. Here, cupric ion was used as an amino acid protecting group during the synthesis keeping the temperature at 0 °C, then it was removed at the end by ethylenediaminetetraacetic acid (EDTA) complexation, generating EDTA-Cu hazardous wastes [10]. From an environmental point of view, our main challenge for the zwitterionic modification of cellulose is to find new procedures to get an appropriate functionalization degree using less demanding and polluting conditions. Recently, we demonstrated the possibility of uptake Congo red dye up to 541.8 mg g^−1^ by means of 16.6% of zwitterionic modification of cellulose with Trimethoxysilylpropyldiethylenetriamine and1,4 butane sultone (Cel-TAS) under a heterogeneous reaction [23]. However, the preparation of the zwitterionic precursor (TAS) was made refluxing the reaction mixture in tetrahydrofuran (THF), which implies temperature and a hazardous solvent.

One approach for cellulose modification that matches this challenge is using activated dicarboxylic amino acid anhydrides as modifiers of cellulose under a less aggressive reaction medium. However, the classical methods for modifying polysaccharides with anhydrides are catalyzed with acetic acid at 85 °C [24] or by aqueous NaOH [25], which can promote either decomposition or depolymerization of cellulose. There have been only few previous reports describing the use of heterogeneous methods for modifying cellulose with anhydrides in less alkaline aqueous conditions [26,27]. In one of them, cellulose was modified by esterification with succinic anhydride and NH_4_OH solution, employing thermal treatments from 160 to 210 °C. Another report involved maleic anhydride in the presence of ammonia (both gaseous NH_3_ and NH_4_OH solution) at room temperature and with reaction times from 1 to 48 h. The result of this work was the formation of cellulose aspartate with a degree of substitution (DS) from 0.001 to 3.0. The authors suggest that the aspartate was also formed in the same esterification process, due to the ammonia addition onto the double bound of the maleic anhydride.

In addition to the latter, this aspartate modification of cellulose seems to be difficult to control due to cross-linking of the polysaccharide through two aspartate residues. Although those methods are environmentally safe for the chemical modification of cellulose, the first involved high temperatures (energy consumption), which can also degrade the polysaccharide matrix. In the second process, reaction times could be too long (up to 48 h), and the amino acid functionality of the aspartate would not always be available, since the amino group could be involved in the cross-linking. Therefore, from our particular point of view, these problems could be avoided by using N-protected amino acid anhydrides, in aqueous ammonia under mild conditions and moderately short reaction times (60 °C/2 h), followed by protecting group cleavage. This main challenge can be achieved introducing a cellulose pretreatment step that could promote better conditions to the amino acids grafting process [28].

Accordingly, in the present work we show a simplified procedure for preparing zwitterionic cellulose, by reacting N-protected aspartic anhydrides with cellulose, which has been previously activated with a minimal amount of NH_4_OH. All steps are taking place under mild conditions and involve less polluting chemical procedures. The structural characterization of the resulting zwitterionic celluloses was carried out by Fourier Transform Infrared with Attenuated Total Reflectance (FTIR-ATR) and ^13^C solid state NMR. The degree of functionalization of the samples was first estimated by ^13^C NMR and corroborated by elemental analysis. Ninhydrin assay [22] was employed to investigate whether or not the reaction conditions also promoted concomitant N-deprotection in the aspartate graft. Finally, the zwitterionic character and the availability of the amino acid residues in cellulose were evaluated by a potentiometric titration curve and dye adsorption test.

## 2. Results and Discussion

### 2.1. Spectroscopic Characterization of the Cel-Asp Samples

**Figure 1:**
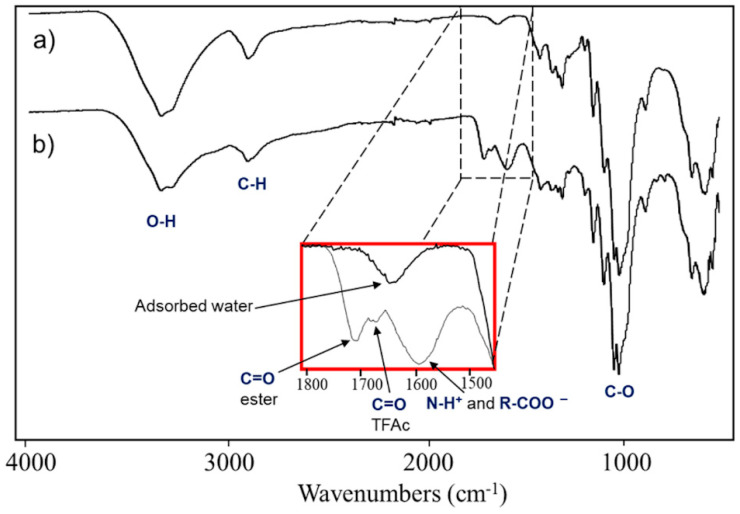
Fourier Transform Infrared spectra of: (**a**) Cellulose sample and (**b**) cellulose modified with N-trifluoroacetyl-L-aspartic anhydride (Cel-Asp-N-TFAc).

Figure 1 shows the FTIR spectra of cellulose (a) and cellulose modified with N-Trifluoroacetyl-L-aspartic anhydride (Cel-Asp-N-TFAc) (spectrum b). In both spectra, typical signals corresponding to the cellulose chemical groups are observed at 3334 and 3276, 2900 and 1053 and 1023 cm^−1^ which are due to O-H, C-H and C-O stretching vibrations, respectively [29,30]. In the sample Cel-Asp-N-TFAc, there are some spectral differences compared to untreated cellulose which are shown in the highlighted inset between 1800 and 1500 cm^−1^. Cellulose spectrum (a) presented the classical band at 1640 cm^−1^ assigned to adsorbed water. In spectrum **b**, new bands confirmed the presence of the aspartic ester grafted onto cellulose. The signal located at 1712 cm^−1^ is assigned to ester carbonyl groups and the small band at 1673 cm^−1^ could be attributed to C=O stretching of the trifluoroacetyl N-protecting group. The last broad and a strong band at 1591 cm^−1^ could be originated by carboxylate groups (R-COO^−^) as well as -N-H^+^ [31,32,33] indicating the presence of the zwitterionic form of the amino acid. On other hand, the signal attributed to the N-H bending vibration of NH-TFAc is overlapped by the carbonyl signal at 1673 cm^−1^.

**Figure 2:**
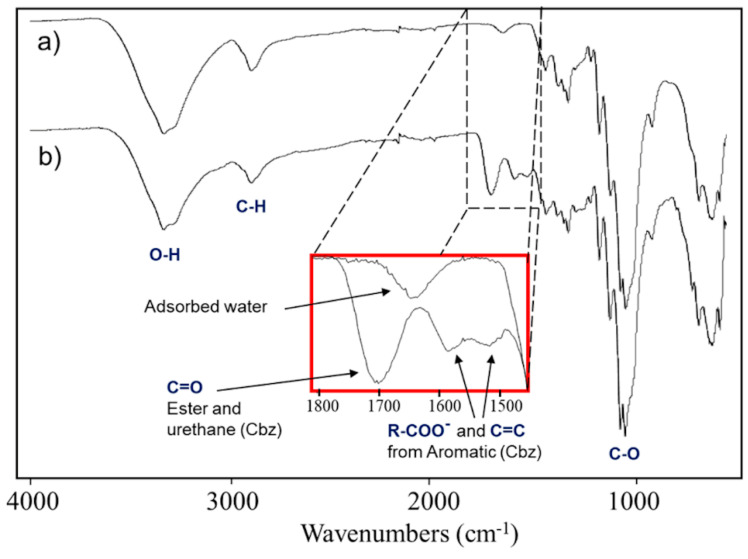
FTIR spectra of: (**a**) cellulose sample and (**b**) cellulose modified with N-carbobenzyloxy-L-aspartic anhydride (Cel-Asp-N-Cbz).

FTIR spectra of cellulose and cellulose modified with N-Carbobenzyloxy-L-aspartic anhydride (Cel-Asp-N-Cbz) are depicted in Figure 2a,b, respectively. Once again, the presence of the cellulose matrix is observed in both samples by the classical O-H, aliphatic C-H and C-O signals pattern as was described previously. A carbonyl signal located at 1700 cm^−1^ is observed clearly in the inset region (1800–1500 cm^−1^) of spectrum **b**, which could arise from the aspartic ester group on cellulose, as well as the urethane moiety of the N-protecting group Cbz. Furthermore, bands at 1588 and 1521 cm^−1^ can be attributed to either carboxylate groups from the aspartic acid or to aromatic rings from Cbz.

**Figure 3:**
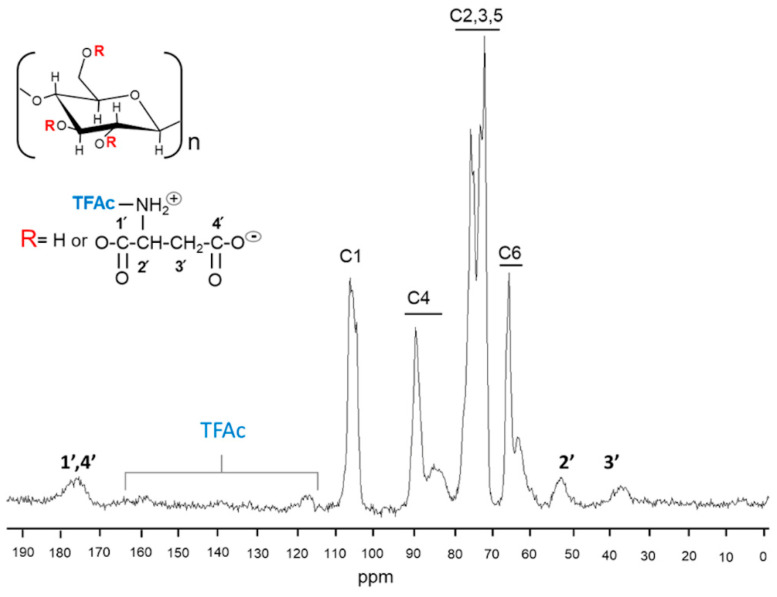
Carbon 13 by cross polarization magic angle spinning (^13^C CPMAS) Nuclear Magnetic Resonance (NMR) spectrum of cellulose modified with N-trifluoroacetyl-L-aspartic anhydride (Cel-Asp-N-TFAc).

**Figure 4:**
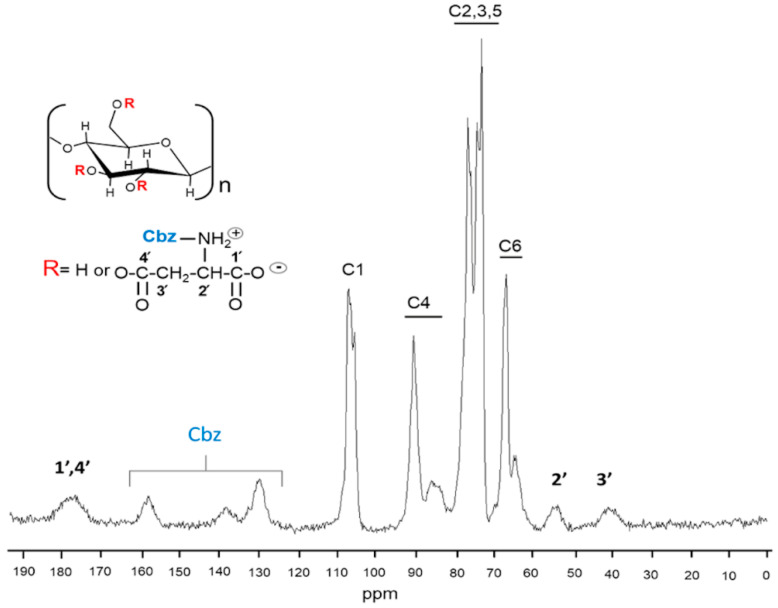
^13^C CPMAS NMR spectrum of cellulose modified with N-carbobenzyloxy-L-aspartic anhydride (Cel-Asp-N-Cbz).

Modified cellulose samples were also analyzed by ^13^C solid state NMR. These spectra are shown in Figure 3 for Cel-Asp-N-TFAc and Figure 4 for Cel-Asp-N-Cbz. ^13^C CPMAS NMR spectra of modified celluloses showed the carbon signals pattern of the cellulose matrix anhydroglucose repeating units (C1 to C6), in the chemical shift range from 60 to 110 ppm [34]. Furthermore, the characteristic signals profile remains for cellulose I (with the crystalline and amorphous components defined in C4 and C6) suggesting that neither the cellulose alkaline pretreatment nor the modification process significantly affects the cellulose crystalline structure (less than 10%) [23,35] as can be observed in Figure 3 and Figure 4. In the spectrum of the Cel-Asp-N-TFAc sample, signals corresponding to the aspartic group grafted on cellulose at 176, 53 and 36 ppm are observed. A broad signal at 176 ppm is attributed to the carbonyl carbons from both the ester and carboxylate moieties and the signals at 53 and 37 ppm are assigned respectively to the α and β carbons of the amino acid [36]. Surprisingly, the small size of signals at 116 and 158 ppm may be attributed to the loss of some Trifluoroacetyl N-protecting group. This fact suggests that unexpected spontaneous deprotection is taking place during the synthesis procedure. In contrast, Figure 4 shows that the carbon signals corresponding to the N-protecting group Cbz in the sample Cel-Asp-N-Cbz, are still clearly observed at 129 and 137 ppm (the aromatic ring carbons). In addition, the carbonyl from the urethane moiety at 157 ppm is present as well. This confirmed that the Cbz group was not cleaved during cellulose modification, while TFAc was extensively removed. Other conclusions can be drawn from the signals corresponding to α and β carbons in the amino acid backbone, located at 52 and 39 ppm, respectively. This last signal, assigned to the beta carbon of the aspartate, seems to be shifted up-field around 2 ppm in sample Cel-Asp-N-TFAc (Figure 3). This fact may indicate differences in amino acid orientation or, in other words, which carbonyl from the aspartic anhydride is connected to cellulose. Since Cbz remains in the modified cellulose Cel-Asp-N-Cbz and this protecting group is bulkier than TFAc, we can picture the scenario where the amino acid (aspartate) is oriented alpha, and the carbonyl carbon **4′** is attached to cellulose as shown in the Figure 4. On the other hand, the amino acid in the Cel-Asp-N-TFAc would be connected to cellulose through carbon **1′** following a beta orientation as isoaspartate (see Figure 3). These changes in the amino acid orientation attached on cellulose can be attributed to the strong electron withdrawing effect on carbon **1′** of the aspartic anhydride due to the TFAc N-protecting group. Therefore, carbon **1′** is more electrophilic when TFAc is used, in comparison with aspartic anhydride with N-Cbz, due to the electron withdrawing inductive effect from the three fluorine atoms alpha to the carbonyl promoting a regioselective reaction, as was recently reported by Sahoo et al. [37]. Additionally, the steric effect of TFAc is smaller than Cbz.

### 2.2. Evaluation of the Functionalization and Deprotection Degrees in Aspartate-Modified Celluloses

Table 1 shows the solid state ^13^C NMR signal integration and Elemental Analysis results, for the grafting degree (GD) in the modified celluloses, using aspartic anhydride with TFAc or Cbz N-protecting groups. When ^13^C NMR was used, the calculations were based on the ratio between the integral values of the cellulose C1 signal and the aspartate alpha carbon (Cα) in each cellulose sample (Cel-Asp-N-TFAc and Cel-Asp-N-Cbz) (see Figures S1 and S2 in the Supporting Material Section). The percentage of functionalization was also determined by fixing the C1 signal area of each sample as 100%. From this calculation, the Cel-Asp-N-TFAc sample showed a moderately higher GD with 18%, while Cel-Asp-N-Cbz was 15%, as can be observed in Table1. Those results are in fairly close agreement with the results from Elemental Analysis (subtracted from Table S1), at 21 and 17%, respectively. These small differences between both methods (2–3%) for quantifying the grafting degree in cellulose support the idea of using NMR spectroscopy not only for characterization but also for a rapid estimation concerning to the degree of functionalization. Besides, these results averaged 20 and 16% in Cel-Asp-N-TFAc and Cel-Asp-N-Cbz, respectively, and are in agreement with the grafting values reported for cellulose in heterogeneous modifications in comparison with the homogeneous procedures [20,38,39,40] as well as the regioselectivity addressed principally to C6 of AGU in esterification reactions [41]. On the other hand, the grafting degree found in the Cel-Asp samples avoids the possibility of zwitterion self-association between vicinal amino acids which could be a problem when the application of the modified cellulose requires active free zwitterionic groups.

Due to the synthesis procedure for sample Cel-Asp-N-TFAc, an important cleavage of the TFAc protecting group occurred (observed in the spectrum shown in Figure 3). The ninhydrin test was applied to both samples (Cel-Asp-N-TFAc and Cel-Asp-N-Cbz) in order to determine the level of deprotection or released amino groups. The degree of deprotection was found by quantitatively determining of the free amino group in the amino acid grafted after the synthesis of both samples of cellulose. In column 6 of Table 1, it can be seen that the free amino group for Cel-Asp-N-TFAc was 13%. This means, that taking in account the average of functionalization degree for this sample of 20%, 65% of this value was N-deprotected (Cel-Asp-N-H). This finding simplifies the two steps of reaction in one. This important conclusion is reached, from the fact that it was shown that during the synthesis process using the anhydride with N-TFAc protecting group, two reaction steps are involved: zwitterionic functionalization and N-deprotection. A plausible explanation of this N-deprotection can be addressed to the nucleophilic attack of the ammonia group from the NH_4_ OH at the electrophilic carbonyl carbon to the TFAc group leaving the alpha amino group of the aspartate residue free. Similar behavior was already reported when ammonia groups are added to the double bounds of the maleic ester in cellulose [27]. In addition, this one-step procedure offers clear advantages in time and chemical consumption when it is compared with similar proposals of modifying cellulose with N-Protected amino acids [22,42].

On the other hand, sample Cel-Asp-N-Cbz did not develop the typical purple color after the addition of ninhydrin, therefore 0% is shown in Table 1. This indicated that all of Cbz N-protecting groups remained in the aspartate graft since it was stable under the reaction conditions used [43]. Again, this confirms the idea that the more electrophilic carbonyl of TFAc N-protecting group, due to the inductive effect of fluorine, may be responsible for the 65% aspartate deprotection currently occurring during the synthesis. Nevertheless, some attempts focused in increasing the N-deprotection in Cel-Asp-N-TFAc were tested under different reaction conditions (NH_4_OH amount, time reaction and temperature). Also, a post-reaction step was introduced where the sample was washing with NH_4_OH/H_2_O at different ratios. However, no progress was achieved that implied higher degree of grafting or N-deprotection. On the contrary, in most of the cases, functionalization degree was the main value affected.

### 2.3. Zwitterion Character in the Cel-Asp-N-H

**Figure 5:**
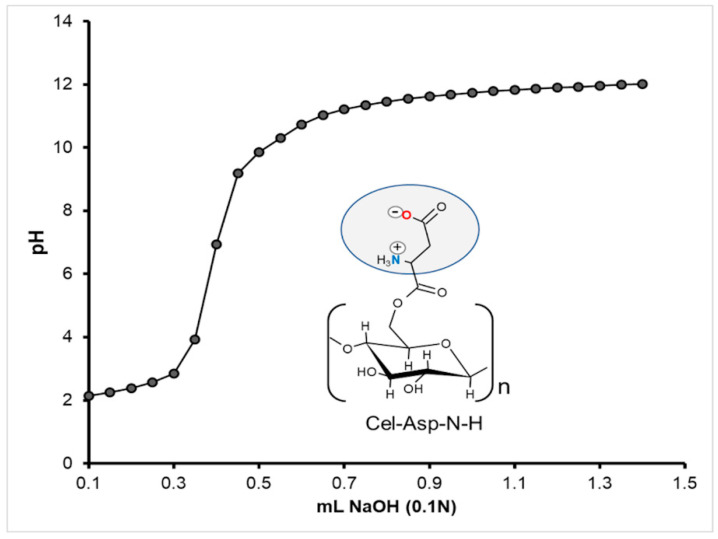
Titration curve of the Cel-Asp-N-deprotected.

Since Cel-Asp-TFAc was the only sample that showed amino group deprotection in the same synthesis process, experiments conducted to evaluate the zwitterionic character (-COO^−^ and -NH_3_^+^) were focused exclusively on it. For this purpose, a titration curve with NaOH 0.1 N was performed to determine the pH value where the amino acid of the sample reaches its isoelectric point or the zwitterion form. Figure 5 depicts the titration curve behavior of the sample Cel-Asp-N-H at different pH values from 2 to 12 vs. mL of NaOH 0.1N added. Here, isoelectric point around pH 6 is established at middle of the pH values between 3 to 9 according to the *pKa* values of the acid and amino groups of the aspartic residue, respectively. These results confirm the zwitterionic functionality conferred to this cellulose sample by means of the grafted amino acids.

### 2.4. Availability of the Zwitterionic Moieties in Cel-Asp-N-H by a Dye Adsorption Test

**Figure 6:**
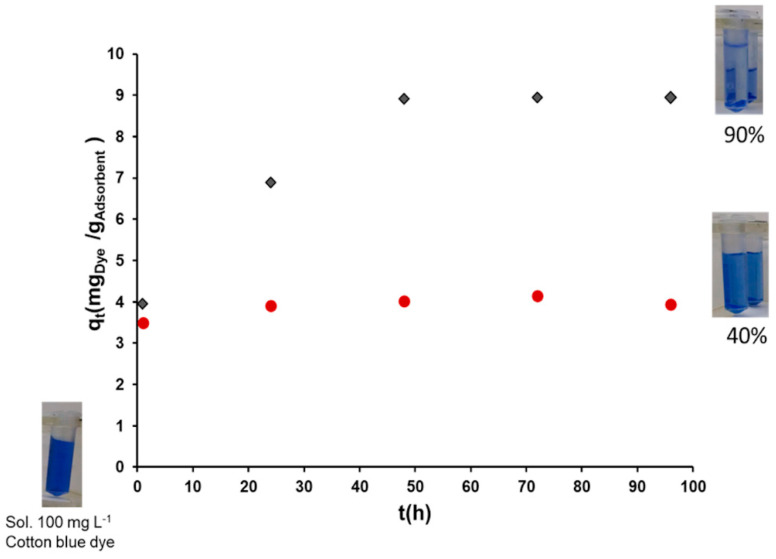
Cotton blue dye adsorption kinetics at pH 5 and 30 °C: Unmodified cellulose (red spheres) and Cel-Asp-N-deprotected (black diamonds).

The ionic availability and activity in the zwitterionic residues of Cel-Asp-N-H sample was evaluated by means of a dye adsorption test. Cotton blue dye is a water-soluble anionic compound due to sulfonate functional groups that remain ionized above a pH value of 3. According to this and the previous determined pH value at which isoelectric neutrality of Asp residues occurs (pH 6), adsorption experiments were conducted into this range at pH 5 which is suggested for dye adsorption in lignocellulosic materials [44]. Under this condition, cotton blue dye shows its anionic form as well as aspartic residues in cellulose present their zwitterion form. Figure 6 shows the adsorption kinetic curve of the cotton blue dye at 100 mg L^−1^ in unmodified cellulose and Cel-Asp-N-H samples up to 94 h. The uptake ability of the materials is observed after 1 h of contact time. Here, the dye adsorption values are 3.5 and 3.9 milligrams per gram in cellulose and Cel-Asp-N-H (mg Dye/g Adsorbent), respectively. These close could be interpreted in the sense that the adsorption mechanism in this first step is mainly attributed to cellulose. This fact is supported by its known intrinsic physical and physicochemical ability to interact with dyes [45]. However, the unmodified cellulose sample reached a maximum of 40% (3.9 mg) of dye uptake after 96 h of contact time. In contrast, functionalized Cel-Asp-N-H showed an enhanced adsorption capacity of up to 70% of cotton blue dye after 24 h, now as a consequence of the ionic interaction mechanism. Moreover, its maximum dye uptake capacity (90%) was achieved after 48 h. This dye uptake value remains after 72 and 96 h of contact time, which confirms that the maximum dye uptake value for the zwitterionic cellulose is reached at 48 h. These results demonstrated that the zwitterionic functionalization of cellulose, in comparison with unmodified cellulose, conferred increased adsorption capacity associated either with their active ionic character as well as the availability of the grafted amino acids. Furthermore, although no separated data were obtained about the performance of the residual 35% of Cel-Asp-N-TFAc in the zwitterionic sample, we cannot neglect the possible contribution of this N-protected sample to the dye adsorption through the coexistence of hydrogen bond interactions. On the other hand, no extensive adsorption studies were performed to this modified cellulose since the scope of the present research was focused to show a simple method to synthesized zwitterionic cellulose as well as a rapid landscape of the benefits of the modification for future applications.

In summary, the present work proved: (1) a simplified process of zwitterionic modification in renewable materials as cellulose, (2) in the same reaction conditions an important amine releasing is taking place when the aspartate anhydride is N-protected with TFAc, (3) a feasible method of modification under mild conditions and less polluted media, and (4) the presence of the ionic dual function in cellulose that could be applied to different areas such as environmental (i.e., the uptake of textile dyes or heavy metal ions in water), biotechnology (i.e., protein or enzyme immobilization), interfacial interaction (adhesives), among others.

## 3. Materials and Methods

### 3.1. Materials

Powdered spruce cellulose was used with a degree of polymerization about 560 (Fluka, Steinheim, Germany). N-Z-L-aspartic anhydride (95%) and (s)-(−)-2-(Trifluoroacetamido) succinic anhydride (97%) came from Sigma-Aldrich Chemie, GmbH, (Darmstadt, Germany). Ammonium hydroxide (28% NH_3_ in H_2_O) was purchased from EMScience filial of Merck KGaA, Darmstadt, Germany. Acetone ACS reagent was supplied from Sigma-Aldrich, S. de R.L. de C.V., Toluca, Mexico and cotton blue dye (C.I. 42755) was bought to Golden Bell, Mexico City, Mexico. All chemicals were used as received.

### 3.2. Preparation of the Zwitterionic Celluloses from Aspartic Anhydrides

**Figure 7:**
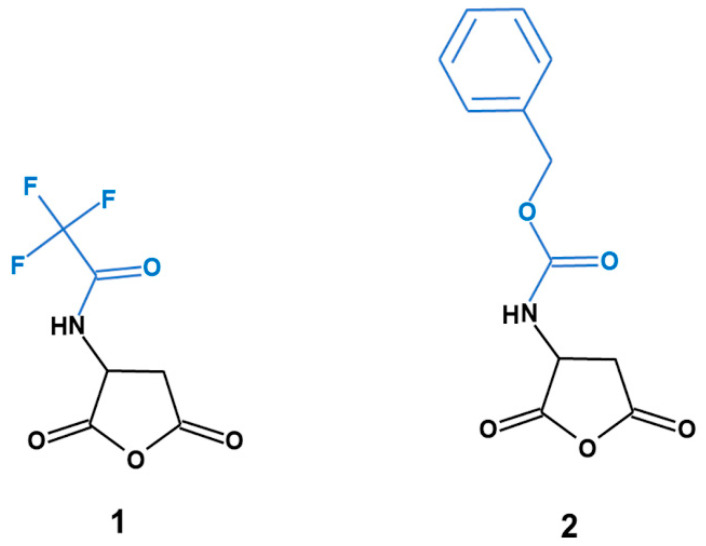
Aspartic anhydrides with N-protective group: (**1**) Trifluoroacetyl (TFAc) and (**2**) carbobenzyloxy (Cbz).

**Figure 8:**
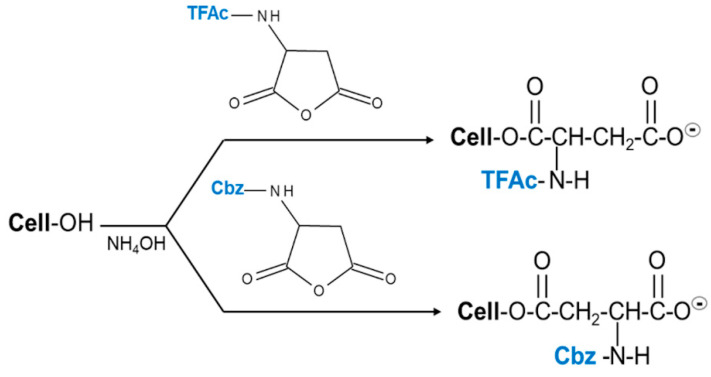
Schematic diagram of the syntheses of cellulose aspartate N-protected with TFAc and Cbz.

Two separate samples of cellulose aspartate (Cel-Asp) were activated by swelling 1.0 g (6 mM) of powdered cellulose in ca. 3 mL of aqueous NH_4_OH (28%), with sporadic stirring, during 15 min at room temperature. After this period, the excess of NH_4_OH was removed by decantation. This treatment was repeated thrice for both samples. Then, one of the activated cellulose samples (1.0 g each) was reacted with 0.38 g (1.8 mM) of (s)-(−)-2-(Trifluoroacetamido) succinic anhydride (N-Trifluoroacetyl-L-aspartic anhydride) (1) dissolved in 2 mL of acetone. Likewise, the second activated cellulose sample (1.0 g) was reacted with 0.45 g (1.8 mM) of N-Z-L-aspartic anhydride (N-Carbobenzyloxy-L-aspartic anhydride) (2) (Figure 7) dissolved in 2 mL of acetone. Then both samples were stirred sporadically at room temperature during 15 min (when the acetone solution was fully absorbed by the cellulose). Thereafter, the mixtures were kept in a fume hood until most of the acetone evaporated, and the smell of ammonia was absent. Then, to complete the chemical modification (Figure 8), the samples were heated in an oven for 2 h at 60 °C. Purification of the crude cellulose aspartates from 1 (Cel-Asp-N-TFAc) and 2 (Cel-Asp-N-Cbz)) was carried out by first washing and filtering them three times with water (5 mL). This washing procedure was repeated in the same manner using acetone. Finally, the clean pale-yellow products were dried in an oven at 60 °C for 4 h.

### 3.3. Characterization

FTIR spectra were obtained by using a Nicolet iS5 Thermo Fisher Scientific FTIR spectrophotometer (81 Wyman Street, Waltham, MA, USA). The samples were directly measured by ATR device using 60 scans to reduce the signal/noise ratio with a 2 cm^−1^ of resolution.

Solid state ^13^C-NMR spectra were acquired using a 14 Tesla Jeol ECA 600 spectrometer (Tokyo, Japan) operated at room temperature, with a rotor spinning speed set to 10 kHz. Samples were packed into a 4 mm I.D. Si_3_N_4_ rotor and measured in a two channels (H, X) solid state DOTY probe. ^13^C CPMAS NMR experiments were used at an operation frequency of 150.9 MHz and a 90° pulse width of about 3.08 µs. The CP contact time was 3 ms with a 10 s of acquisition delay.

### 3.4. Determination of the Degree of Functionalization in Celluloses

The degree of functionalization or grafting degree (GD) in celluloses was determined quantitatively by elemental analysis (EA 1108 Fisons Instrument model EA1108 CHNS, distributed by Thermo Fisher, Waltham, MA, USA) calculating the C/N ratio. Comparatively, solid state ^13^C NMR was also used to estimate the functionalization [46,47,48,49,50,51]. Thus, by inspection of the ^13^C-NMR spectrum of each sample, the integral value of the signal corresponding to C1 of AGU in cellulose (~106 ppm) [34] was used as reference. This value was compared with the integral value of the **α** carbon (Cα) signal of the N-TFAc and N-Cbz-aspartate grafts.

### 3.5. Determination of the N-Deprotection Degree of the Aspartate

The amount of N-deprotection in the aspartate grafts in the modified celluloses was measured by using a ninhydrin method previously reported [22]. Celluloses with N-deprotected aspartates react with a ninhydrin solution developing a blue-purple color due to Schiff base formation [52]. The deprotection level in each sample was calculated by comparing the UV absorbance values at 575 nm, with the values from a calibration curve of valine at different concentrations (2, 4, 6, 8 and 10 mM).

### 3.6. Zwitterion Character Evaluation

The evaluation of the zwitterion character or the isoelectric point in the modified cellulose samples was inspected by the classical potentiometric titration method. The criterion for this evaluation is based on determining the protonation-deprotonation process preferentially in modified celluloses with free amine and acid functional groups. Briefly, a solution of 20 mg of N-deprotected sample in 20 mL of acidified water (pH 2) is titrated by the addition of small aliquots of 0.1 mL of NaOH 0.1N until constant pH value. The titration curve was obtained by recording the pH values after adding each NaOH volume.

### 3.7. Availability of the Zwitterionic Moieties by Dye Adsorption Test

In order to evaluate the ionic contribution in the zwitterionic cellulose samples avoiding possible steric hindrance of the protecting groups in the amine, a simple adsorption test is performed only for N-deprotected samples. A triplicated experiment of 15 mg of modified cellulose sample in Eppendorf tubes (2 mL), previously treated with buffer of acetates (pH 5), were treated with 1.5 mL of a 100 mg L^−1^ solution of cotton blue dye (ionic dye), which is also buffered to pH 5. The same treatment is applied to unmodified cellulose sample as a control. The tubes with the mixtures were constantly stirred at 100 rpm in a laboratory incubator shaker at 30 °C. Dye adsorption capacity of the modified cellulose was determined at different contact time (1, 24, 48, 76 and 96 h). After each contact time, samples in the tubes were centrifugated at 10,000 rpm/5 min and the concentration of the residual cotton blued dye of the supernatant solution was measured at 595 nm (λ_max_) in a UV-Vis spectrometer Varian CARY 50CONC.

Dye adsorbed amount per unit of adsorbent *q_t_* (mg g^−1^) was calculated applying Equation (1):

(1) $$q_{t}=\frac{\left(C_{0}-C_{t}\right)V}{W}$$

where, *C_0_* and *C_t_* are initial concentration of dye and concentration of the residual dye after time *t*, mg L^−1^; *V* is the volume of the solution, (L), and *W* is the weight of modified cellulose used as adsorbent (g).

## 4. Conclusions

This investigation demonstrated the feasibility of synthesizing two zwitterionic celluloses Cel-Asp-N-TFAc and Cel-Asp-N-Cbz, through a simple and heterogeneous reaction procedure, using commercially available N-protected aspartic anhydrides, employing mild aqueous conditions and a minimal use of organic solvents, namely acetone. The functionalization degree attained for cellulose functionalized with TFAc and Cbz N-protected aspartic anhydrides was 20 and 16%, respectively. These relatively low GD values also avoid amino acid hindrance due to the association between neighboring zwitterions, which can have a positive effect for applications such as adsorbent. During the synthesis procedure, the Cel-Asp-N-TFAc sample was concurrently N-deprotected up to 65%. In contrast, no purple color was developed, indicating that N-Cbz protecting groups in Cel-Asp-N-Cbz, were not affected during the synthesis. These results demonstrate clear advantages based on the election of amino acid anhydrides N-protected with TFAc compared to Cbz. The zwitterionic character of the Cel-Asp-N-H (N-deprotected) sample was demonstrated by its isoelectric point at pH 6 using a potentiometric titration method. The activity and availability of the zwitterion residues in the Cel-Asp-N-H were evaluated by means of adsorption dye test. Here, a maximum 90% of 100 mg L^−1^ of cotton blue dye solution was adsorbed in Cel-Asp-N-H after 48 h in comparison with the 40% shown in unmodified cellulose at the same contact time.

Finally, the synthesis procedure reported here provides important features; it: (1) is simple to perform, (2) requires minimal energy and time consumption, (3) involves a simultaneous process of zwitterionic modification of cellulose and extensive N-deprotection of the amino acid graft, (4) uses aqueous ammonia and acetone in small amounts in the reaction, implying minimal environmental risk [10,53] and (5) the active zwitterionic form in the modified cellulose could be applied for several purposes, especially where the ionic interaction needs to be implied.

## Figures and Tables

**Table 1:** Percentage of cellulose modification determined by ^13^C NMR and elemental analysis. Column 6 shows the deprotection degree (%) in aspartic residues evaluated by ninhydrin method (details about these calculations are described in Supplementary Material by means of Table S2 and Figure S3).

Modified Celluloses	^13^C NMR Areas		% of mod. by NMR^a^	Elemental Analysis		% of mod. by AE	% of Deprotection in Asp.
	C1	Cα		%C	%N		
Cel-Asp-N-TFAc	58.8	10.4	18%	39.756	1.402	21%	13%
Cel-Asp-N-Cbz	52.0	7.7	15%	44.752	1.398	17%	0%

^a^ Through the integration of the areas from C1cellulose vs. Cα of Aspartic residue.

## Supplementary Materials

The following are available online, Figure S1: ^13^C CPMAS NMR spectrum of Cel-Asp-N-TFAc with integral values for each signal, Figure S2: ^13^C CPMAS NMR spectrum of Cel-Asp-N-Cbz with integral values for each signal, Figure S3: Calibration curve of valine at different concentrations: (1) experimental data (continuous line) and (2) calculated (dashed line). Table S1: Elemental analysis data from samples of Cel-Asp-N-TFAc and Cel-Asp-N-Cbz compared with their theoretical functionalization degrees, Table S2: Absorbance values for valine solutions with different concentrations were used for the calibration curve.
