# Supplementary material for: A Simplified Method of Synthesis to Obtain Zwitterionic Cellulose under Mild Conditions with Active Ionic Moieties
Source: Molecules. 2020 Jul 5;25(13):3065. doi: 10.3390/molecules25133065 (PMC7412472; doi:10.3390/molecules25133065)
Supplement: Supplementary file 1 [file molecules-25-03065-s001.pdf]

molecules

MDPI

Supplementary Material

A Simplified Method of Synthesis to Obtain Zwitterionic Cellulose under Mild Conditions with Active Ionic Moieties

Nadia B. Haro-Mares, Juan C. Meza-Contreras, Fernando A. López-Dellamary Toral, Ricardo González-Cruz, José A. Silva-Guzmán and Ricardo Manríquez-González *

Department of Wood, Cellulose and Paper, CUCEI, University of Guadalajara, 45020 Guadalajara, Jalisco, Mexico; aradia_b@hotmail.com (N.B.H.-M.); jcmezac@academicos.udg.mx (J.C.M.-C.); ferdellam@gmail.com (F.A.L.-D.T.); ricardo.gonzalezc@academicos.udg.mx (R.G.-C.); jasilva@dmcyp.cucei.udg.mx (J.A.S.-G.)

* Correspondence: rmanri@dmcyp.cucei.udg.mx and rmanri@gmail.com; Tel.: +52-33-3682-0110

1. Experimental

1.1. Measurement of the Grafting Degree of Cellulose

1.1.1. Nuclear Magnetic Resonance Analysis

The estimation of the grafting degree (GD) in cellulose was calculated from the spectra of the samples of cellulose modified with N-protected aspartic anhydride. The protecting groups were trifluoroacetyl (Cel-Asp-N-TFAc) and carbobenzyloxy (Cel-Asp-N-Cbz), as shown in Figures S1 and S2, respectively. For each spectrum, the integration value of the C1 of the anhydroglucose unit (AGU) of cellulose (~106 ppm) was compared with that of the alpha carbon ($C\alpha$) of the grafted aspartate residue, in each sample (52–53 ppm), as can be observed in both spectra (Figure S1 and S2). Furthermore, to provide assurance that the integration values are trustworthy, all the signals have their integration values depicted. The integration values for each carbon in cellulose (110–60 ppm) should be similar, as should be those belonging to the aspartic acid graft [1]. Thus, the signals corresponding to the aspartic graft as beta carbon (C_β) should have similar integration values to those of the neighboring C_α, in order to consider that the NMR experimental parameter chosen were appropriate. In the case of the spectrum of sample Cel-Asp-N-TFAc, the cellulose carbon integration value had an average of 57 and that for the aspartate C_α and C_β was around 10.3. Carbon signal integration areas for Cel-Asp-N-Cbz were around 52.5 for cellulose and an averaged value of 7.7 for the aspartic carbons. Accordingly, the reference carbon area was assigned as 100% to that belonging to the C1 of the AGU of cellulose, so the ratio of this value vs the integration value $C\alpha$ of the aspartate in each sample was used to calculate the degree of substitution.

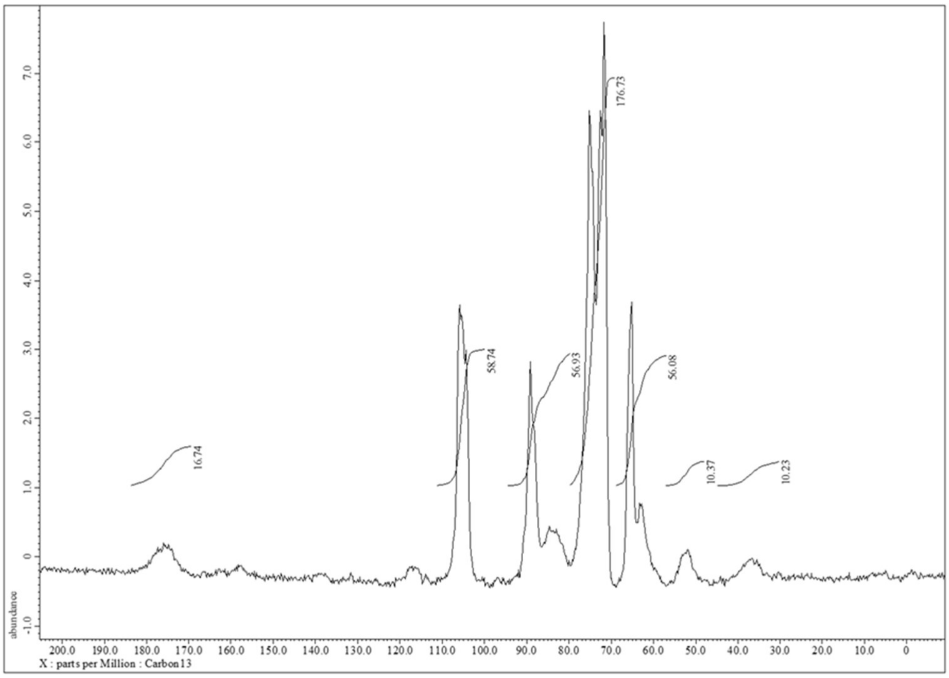

Figure S1. ^{13}C CPMAS NMR spectrum of Cel-Asp-N-TFAc with integral values for each signal.

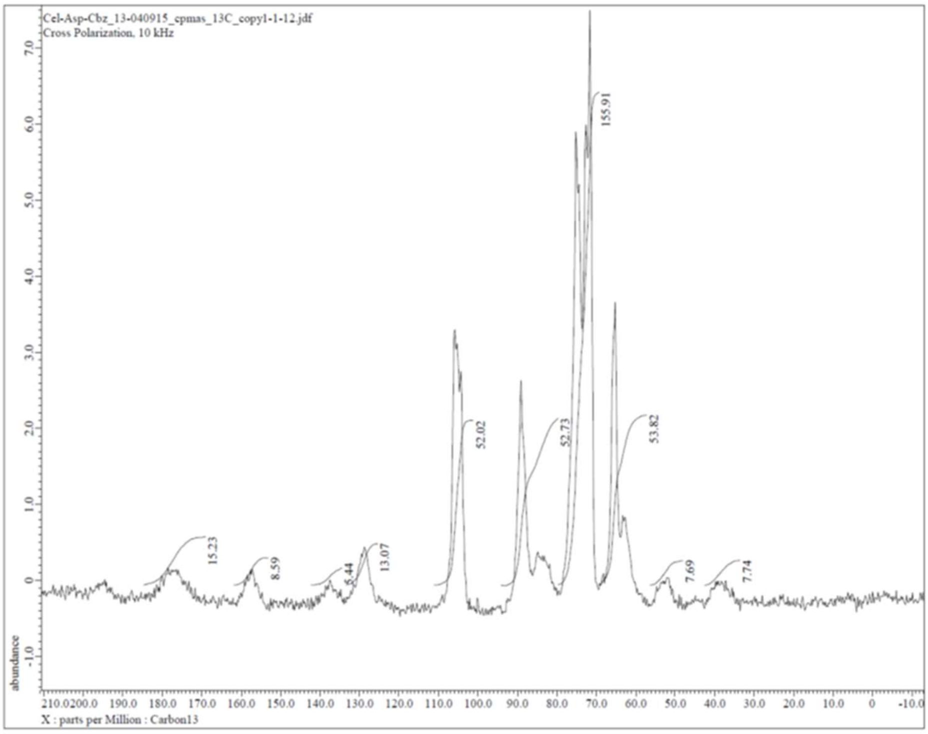

Figure S2. ^{13}C CPMAS NMR spectrum of Cel-Asp-N-Cbz with integral values for each signal.

1.1.2. Elemental Analysis (EA)

Elemental analysis was carried out with around 2 mg of each sample (Cel-Asp-N-TFAc and Cel-Asp-N-Cbz) in duplicated using a Fisons Instrument model EA1108 for Carbon, Hydrogen, Nitrogen and Sulfur (CHNS). The percentage of C, N and H of each sample was determined and averaged to calculate

the GD in cellulose. Table S1 shows the resulting data. Here the experimental data from EA of cellulose [2] and modified cellulose are comparable with theoretical values according to the degree of substitution calculated in base of the relationship of AGU´s of cellulose vs aspartate residue. The Cel-Asp-N-TFAc sample shows a ratio of 5:1 anhydoglucose units per aspartate residues, calculated from the C and N content, corresponding to a functionalization (GD) of 20%. In the case of Cel-Asp-N-Cbz a 17% of GD was obtained and is related to the 6:1 ratio of AGU and aspartate residues.

Table S1. Elemental analysis data from samples of Cel-Asp-N-TFAc and Cel-Asp-N-Cbz compared with their theoretical functionalization degrees.

Sample	Run 1			Run 2			Average		
	%C	%N	%H	%C	%N	%H	%C	%N	%H
Cellulose	42.60		7.15	42.57		7.14	42.59		7.14
Cellulose	*Calculated according to cellobiose unit ($C_{12}H_{22}O_{11}$)*						*42.10*		*6.42*
Cel-Asp-N-TFAc	40.91	1.41	4.83	41.03	1.37	4.93	40.97	1.39	5.83
AGU/Asp-N-TFAc (5:1)	*Calculated according to 20% of grafting degree ($C_{36}H_{54}F_3NO_{29}$)*						*40.89*	*1.35*	*5.33*
Cel-Asp-N-Cbz	46.65	1.30	6.31	46.61	1.09	6.12	46.63	1.19	6.21
AGU/Asp-N-Cbz (6:1)	*Calculated according to 17% of grafting degree ($C_{48}H_{71}NO_{35}$)*						*47.13*	*1.14*	*8.10*

1.2. N-Deprotection Degree on Celluloses

Celluloses with N-deprotected aspartates react with a ninhydrin solution developing a blue-purple color due to the Schiff base formation [3]. The deprotection degree in each sample was measured using a UV/Vis spectrophotometer and calculated by reading the absorbance values at 575 nm, employing a calibration curve with different concentrations of amino acid valine (2, 4, 6, 8 and 10 mM) following a modified Moore–Steain methodology [4]. Five hundred μL of valine solution were placed in 5 mL test tubes and then reacted with 1500 μL of 0.5% ninhydrin solution, capped and heated in a water bath at 95 °C during 15 min. After this period, the tubes were cooled to room temperature with tap water. Each tube developed a blue-purple color with different intensities according to its valine concentration (5 to 10 mM). 100 μL of the colored solution were withdrawn from each tube and bought up to 1 mL with 900 μL of distilled water and transferred to a plastic cuvette (for visible spectroscopy). Every solution was measured at 575 nm using distilled water as blank. The absorbance values, as shown in Table S2, were used to construct a calibration curve (Figure S3). Linear regression analysis was applied to fit the curve, using the equation $y = mx-b$, where y is the equipment response, m is the slope, c the concentration of the sample and b corresponded to the intercept [5]. Furthermore, squared correlation coefficient, R^2, was determined by linear regression analysis in order to determine how well the model fits the experimental data. The closer the R^2 value to 1.0, the better the data points are described by linear regression [6]. In our case, R^2 was resulted in 0.9907 as can be seen in the calibration curve shown in Figure S2.

Table S2. Absorbance values for valine solutions with different concentrations used for the calibration curve.

Concentration (mM)	Absorbance
2	0.0687
4	0.2731
6	0.6049
8	0.9947
10	1.2469

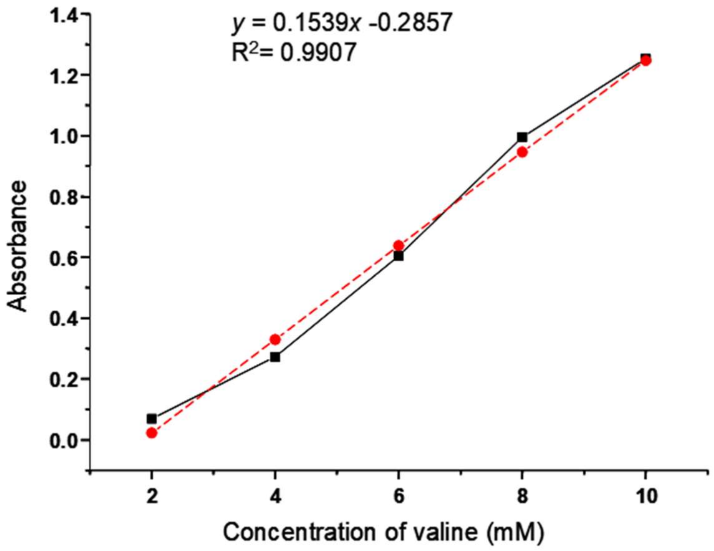

Figure S3. Calibration curve of valine at different concentrations: **1**) experimental data (continuous line) and **2**) calculated (dashed line).

In the case of modified celluloses, 5 mg of each sample (Cel-Asp-N-TFAc and Cel-Asp-N-Cbz) were suspended in 500 μL of distilled water and reacted with 1500 μL of 0.5% ninhydrin solution, in a 5 mL screw-capped tubes. Placed in a water bath at 95 °C for 20 min, with periodic shaking during 3 sec using a vortex after every 5 min. Again, the tubes were cooled at room temperature with tap water, after which 100 μL of the supernatant were taken and transferred to a visible cuvette using 900 μL of distilled water. Clear differences were observed between both modified samples, since the Cel-Asp-N-TFAc was the only sample which developed blue-purple color solution with ninhydrin, in comparison to the Cel-Asp-N-Cbz, without color and having similar absorbance value as the blank (distilled water) when measured at 575 nm. Analysis of Cel-Asp-N-TFAc was performed by triplicate and the absorbance values (0.7715, 0.8444 and 0.9139) resulting and average of 0.8433. This was used to calculate the degree of N-deprotection in aspartic residue in cellulose according to equations (1) and (2):

$$C_{Asp} = \frac{y+b}{m} * \frac{v}{1{,}000{,}000} \quad (1)$$

Where C_{Asp} is the amount in mM of the aspartate residue N-deprotected, y is averaged absorbance of the sample of Cel-Asp-N-TFAc (0.8433), b is the intercept (0.2857 mM) and m the slope of the curve (0.1539) and v is the volume of the sample in μL (500 μL).

$$C_{AGU} = \left[w_{Samp} - \left(C_{Asp} * Mw_{Asp}\right)\right] * \frac{1}{Mw_{AGU}} \quad (2)$$

In this equation 2, C_{AGU} corresponds to the total amount in mM of AGU´s in cellulose, W_{Samp} is weight of the sample (5 mg), MW_{Asp} and MW_{AGU} are the molecular weight of aspartate N-deprotected (116 mg/mM) and anhydroglucose unit (AGU) of cellulose (162 mg/mM), respectively.

Replacing the experimental values in the equations 1 and 2 the resulted vales are:

C_{Asp} = 0.003668 mM of Aspartate

C_{AGU} = 0.0282 mM of AGU´s.

The grafting degree is calculated by the ratio of the C_{Asp}/C_{AGU}, which corresponded to 0.1299 ~ 0.13. In other words, the functionalization of aspartate N-deprotected (Cel-Asp-N-H) is 13%. According to ^{13}C

NMR and EA of the Cel-Asp-N-TFAc sample, the total degree of substitution was 20% and the 13% of this grafted aspartate is N-H. It means that N-deprotection occurred in the same process of modification of cellulose reaching up to 65%.

2. References

1. Okushita, K.; Komatsu, T.; Chikayama, E. and Kikuchi, J. Statistical approach for solid-state NMR spectra of cellulose derived from a series of variable parameter. *Polym. J.* **2012**, *44*, 895–900, doi:10.1038/pj.2012.82.
2. Konno, K.; Onodera, H.; Murata, K.; Onoe, K.; Yamaguchi, T. Comparison of Cellulose Decomposition by Microwave Plasma and Radio Frequency Plasma. *Green and Sustainable Chemistry*, **2011**, *1*, 85–91, doi:10.4236/gsc.2011.13014.
3. Friedman, M. Applications of the ninhydrin reaction for analysis of amino acids, peptides, and proteins to Agricultural and Biomedical Sciences. *J. Agric. Food Chem.* **2004**, *52*, 385−406, doi:10.1021/jf030490p.
4. Moore, S. and Steain, W. A Modified Ninhydrin Reagent for the Photometric Determination of Amino Acids and Related Compounds. *J. Biol. Chem.* **1954**, *211*, 907–913.
5. Skoog, D. A.; Holler, F. J.; Crouch, S. R. Principles of Instrumental Analysis. 6th ed.; Pacific Grove Calif.: Brooks Cole Publishers, London, United Kingdom 2007; pp. 367–398.
6. Sonawane, S.S., Chhajed, S.S., Attar, S.S. *et al.* An approach to select linear regression model in bioanalytical method validation. *J Anal Sci Technol.* **2019**, *10*, 1–7, doi:10.1186/s40543-018-0160-2.
